# Exploiting Semantic Annotations and *Q*-Learning for Constructing an Efficient Hierarchy/Graph Texts Organization

**DOI:** 10.1155/2015/136172

**Published:** 2015-01-01

**Authors:** Asmaa M. El-Said, Ali I. Eldesoky, Hesham A. Arafat

**Affiliations:** Department of Computers and Systems, Faculty of Engineering, Mansoura University, Mansoura, Egypt

## Abstract

Tremendous growth in the number of textual documents has produced daily requirements for effective development to explore, analyze, and discover knowledge from these textual documents. Conventional text mining and managing systems mainly use the presence or absence of key words to discover and analyze useful information from textual documents. However, simple word counts and frequency distributions of term appearances do not capture the meaning behind the words, which results in limiting the ability to mine the texts. This paper proposes an efficient methodology for constructing hierarchy/graph-based texts organization and representation scheme based on semantic annotation and *Q*-learning. This methodology is based on semantic notions to represent the text in documents, to infer unknown dependencies and relationships among concepts in a text, to measure the relatedness between text documents, and to apply mining processes using the representation and the relatedness measure. The representation scheme reflects the existing relationships among concepts and facilitates accurate relatedness measurements that result in a better mining performance. An extensive experimental evaluation is conducted on real datasets from various domains, indicating the importance of the proposed approach.

## 1. Introduction

As the sheer number of textual documents available online increases exponentially, the need to manage these textual documents also increases. This growth of online textual documents plays a vital role in exploring information and knowledge [1]. The massive volume of available information and knowledge should be discovered, and the tasks of managing, analyzing, searching, filtering, and summarizing the information in documents should be automated [1, 2]. Four main aspects pertain to most of the textual document mining and managing approaches: (a) representation models [3], (b) relatedness measures [4], (c) mining and managing processes [2], and (d) evaluation methods.

Selecting an appropriate data representation model is essential for text characterization including text mining and managing, dictating how data should be organized, and what the key features are. The “relatedness measures” are used to determine the closeness of the objects in the representation space, while the “mining and managing processes” are the algorithms that describe the steps of a specific task to fulfill specific requirements. The evaluation methods are used to judge the quality of the mining process results that are produced [1].

**Figure 1:**
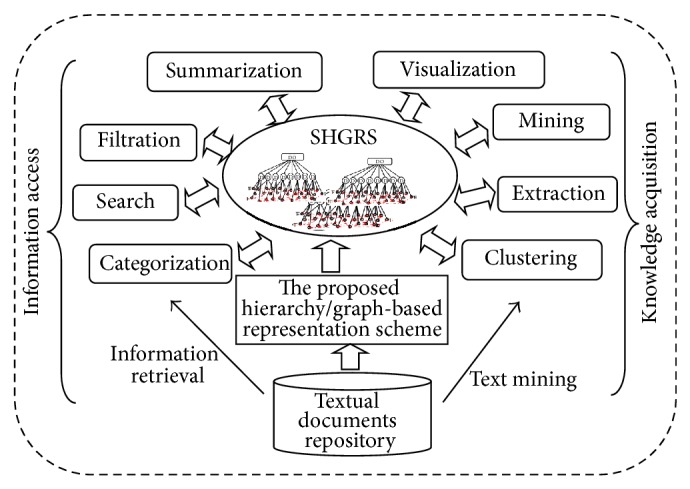
The proposed semantic text organization/representation scheme for information access and knowledge acquisition processes.

At a certain level of simplicity, such mining and managing operations as gathering, filtering, searching, retrieving, extracting, classifying, clustering, and summarizing documents seem relatively similar. All of these operations make use of a text representation model and a relatedness measure to perform their specific tasks. Dealing with natural language documents requires an adequate text representation model to understand them [5]. Accordingly, the trend toward reliance on a semantic understanding-based approach is necessary. Knowledge-rich representations of text combined with accurate semantic relatedness measures are required. This paper introduces an efficient semantic hierarchy/graph-based representation scheme (SHGRS) based on exploiting the semantic structure to improve the effectiveness of the mining and managing operations. Therefore, document clustering in this paper is used as a case study to illustrate the working and the efficacy of the proposed representation scheme. The semantic representation scheme is a general description or a conceptual system for understanding how information in the text is represented and used. The proposed representation scheme as shown in Figure 1 is an essential step for the actual information access and knowledge acquisition. As revealed by the figure below, an effective textual static structure with dynamic behavior is provided to evolve processes for information access and knowledge acquisition. The main contributions of this paper are summarized as follows:

1. proposing an efficient representation scheme called SHGRS that relies on an understanding-based approach;
2. exploiting the knowledge-rich notion to introduce an efficient semantic relatedness measure at a document level;
3. suggesting a novel linkage process for documents through identifying their shared subgraphs at a corpus level;
4. conducting extensive experiments on real-world datasets to study the effectiveness of the proposed representation scheme along with the proposed relatedness measure and linkage process that allow more effective document mining and managing processes.

The rest of the paper is organized as follows.  Section 2 gives a brief overview of the related work and basic concepts while Section 3 illustrates the details about the proposed framework for constructing an efficient text representation scheme based on semantic annotation.  Section 4 reports the experimental results and discussions. Conclusions and suggestions for future work are given in Section 5.

## 2. Basic Concepts and Related Works

In an effort to keep up with the tremendous growth of the online textual documents, many research projects target the organization of such information in a way that will make it easier for the end users to find the information they want efficiently and accurately [1]. Related works here can roughly be classified into three major categories of studies: text representation model, semantic relatedness measures, and documents linkage process.

Firstly, the growing amount of recent research in this field focuses on how the use of semantic representation model is beneficial for text categorization [3], text summarization [8], word sense disambiguation [9], methods for automatic evaluation of machine translation [10], and documents classification [11]. Although the text representation model is a well-researched problem in computer science, the current representation models could be improved with the use of external knowledge that is impossible to extract from the source document itself. One of the widely used sources of external knowledge for the text representation model is WordNet [12], a network of related words organized into synonym sets, where these sets are based on the lexical underlying concept. Furthermore, machine learning is not yet advanced enough to allow large-scale extraction of information from textual document without human input. Numerous semantic annotation tools [13, 14] have been developed to aid the process of human text markup to guide machines. The main task in the text representation model includes morphological, syntactic, and semantic analysis that uses the results of the low-level analysis as well as dictionary and reference information to construct a formalized representation of NL text. Semantic level implies not only linguistic but also logical relations between language objects to be represented [15]. In the semantic level of understanding of text documents [5], models such as the WordNet-based semantic model, conceptual dependence model, semantic graph model, ontology-based knowledge model, and Universal Networking Language can be used. In [16], the WordNet-based semantic model captures the semantic structure of each term within a sentence and document rather than the frequency of the term within a document only. This model analyzes the terms and their corresponding synonyms and hypernyms on the sentence and document levels, ignoring dependencies among terms in the sentence level. In [17], the conceptual dependence model identifies, characterizes, and understands the effect of the existing dependencies among the entities in the model, considering only nouns as a concept. The semantic graph model proposes semantic representation based on a graph-based structure with a description of text structure [5]. In the semantic graph model, the traditional method to generate the edges between two nodes in the graph is usually based on the cooccurrence and similarity measures of two nodes, and the most frequent word is not necessarily chosen to be important. In [18], ontology knowledge represented by entities connected by naming relationships and the defined taxonomy of classes may become a much more powerful tool in information exploration. Traditional text analysis and organization methods can also be enriched with the ontology-based information concerning the cooccurring entities or whole neighborhoods of entities [19]. However, automatic ontology construction is a difficult task because of the failure to support order among the objects and the attributes. In [20], Universal Networking Language (UNL) is used to represent every document as a graph with concepts as nodes and relations between them as links, ignoring the sequence and orders of concepts in a sentence at the document level.

Secondly, the semantic relatedness measure has become an area of research as one of the hotspots in the area of information technology. Semantic relatedness and semantic similarity are sometimes confused in the research literature, and they are not identical [21, 22]. The semantic relatedness measures usually include various types of relationships such as hypernym, hyponym, subsumption, synonym, antonym, holonym, and meronymy. The semantic similarity is a special case of semantic relatedness that only considers synonymous relationships and subsumption relationships. There are several measures for semantic relatedness recently conducted. According to the parameters used, they can be classified into three major categories, including distance-based methods (Rada and Wu and Palmer [21]) which selects the shortest path among all the possible paths between concepts to be more similar, information-based methods (Resnik, Jiang and Conrath, and Lin [21]) which considers the use of external corpora avoiding the unreliability of path distances and taxonomy, and hybrid methods (T. Hong and D. smith and Zili Zhou [21]) which combines the first two measures. In this paper, the information-based methods are focused on. In [23], the semantic relatedness of any concept is based on a similarity theorem in which the similarity of two concepts is measured by the ratio of the amount of information needed to the commonality of the two concepts to the amount of information needed to describe them. The information content (IC) of their lowest common subsumer (LCS) captures the commonality of the two concepts and the IC of two concepts themselves. The lowest common subsumer (LCS) is the most specific concept, which is a shared ancestor of the two concepts. The pointwise mutual information (PMI) [24, 25] is a simple method for computing corpus-based similarity of words. The pointwise mutual information is defined as the ratio of the probability that the two words cooccur *p*(*w*_1_  and  *w*_2_) and the product of the probability cooccurs of each one *p*(*w*_1_) · *p*(*w*_2_). If *w*_1_ and *w*_2_ are statistically independent, then the probability that they cooccur is given by the product *p*(*w*_1_) · *p*(*w*_2_). If they are not independent and they have a tendency to cooccur, then *p*(*w*_1_  AND  *w*_2_) will be greater than *p*(*w*_1_) · *p*(*w*_2_). As it is clear, there is extensive literature on measuring the semantic relatedness between long texts or documents [7], but there is less work related to the measurement of similarity between sentences or short texts [26, 27]. Such methods are usually effective when dealing with long documents because similar documents will usually contain a degree of cooccurring words. However, in short documents, the word cooccurrence may be rare or even null. This is mainly due to the inherent flexibility of natural language enabling people to express similar meanings using quite different sentences in terms of structure and word content.

Finally, in order to overcome the limitations of previous models, the documents linkage process is conducted relying on graph-based document representation model [4, 10, 11]. The main benefit of graph-based techniques is that they allow us to keep inherent structural information of original document. Mao and Chu [10] used graph based *K*-means algorithm for clustering web documents. Yoo and Hu [28] represent a set of documents as bipartite graphs using domain knowledge in ontology. Then documents are clustered based on average-link clustering algorithm. However, these approaches relying on graph-based model are not intended to be used in an incrementally growing set of documents.

To utilize the structural and semantic information in the document in this paper, a formal semantic representation of linguistic input is introduced to build an SHGRS scheme for the documents. This representation scheme is constructed through the accumulation of syntactic and semantic analysis outputs. A new semantic relatedness measure is developed to determine the relatedness among concepts of the document as well as relatedness between contents of the documents for long/short texts. An efficient linkage process is required to connect the related documents by identifying their shared subgraphs. The proposed representation scheme along with the proposed relatedness measure and linkage process is believed to enable a more effective document mining and managing processes.

## 3. The Proposed Semantic Representation Scheme and Linkage Processes

In this section, a new framework is introduced for constructing the SHGRS and conducting the document linkage processes using multidimensional analysis and primary decision support. In fact, the elements in a sentence are not equally important, and the most frequent word is not necessarily the most important one. As a result, the extraction of text main features (MFs) as concepts as well as their attributes and relationships is important. Combinations of semantic annotation [13, 14] and reinforcement learning (RL) [29] techniques are used to extract MFs of the text and to infer unknown dependencies and relationships among these MFs. The RL fulfills sequential decision-making tasks with long-run accumulated reward to achieve the largest amount of interdependence among MFs. This framework focuses on three key criteria: (1) how the framework refines text to select the MFs and their attributes, (2) what learning algorithm is used to explore the unknown dependencies and relationships among these MFs, and (3) how the framework conducts the document linkage process.

**Figure 2:**
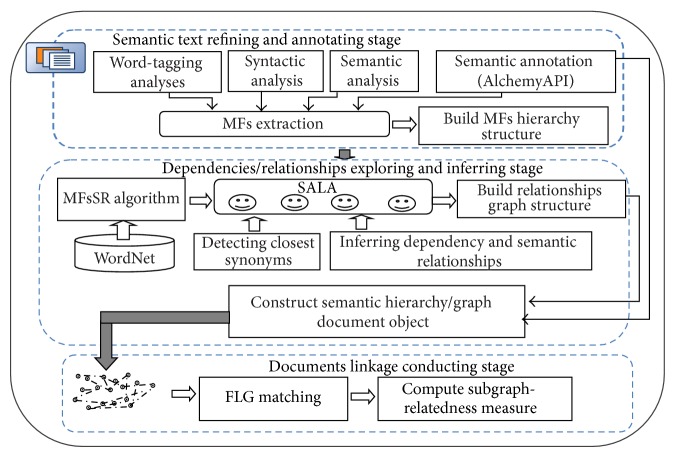
A framework for constructing a semantic hierarchy/graph text representation scheme.

Details of this framework process in three stages are given in Figure 2. The first stage aims to refine textual documents to select the MFs and their attributes with the aid of OpenNLP [13] and AlchemyAPI [14]. Hierarchy-based structure is used to represent each sentence with its MFs and their attributes which achieves dimension reduction with more understanding. The second stage aims to compute the proposed MFs semantic relatedness (MFsSR) that contributes to the detection of the closest synonyms of the MFs and to inferring the relationships and dependencies of the MFs. Graph-based structure is used to represent the relationships and dependencies among these MFs which achieves more correlation through many-to-many relationships. The third stage aims to conduct the document linkage process using a subgraph matching process that relies on finding all of the distinct similarities common to the subgraph. The main proceedings of this framework can be summarized as follows:

1. extracting MFs of sentences and their attributes to build the hierarchy-based structure for efficient dimension reduction;
2. estimating a novel semantic relatedness measure with consideration of direct relevance and indirect relevance between MFs and their attributes for promising performance improvements;
3. detecting closest synonyms of MFs for better disambiguation;
4. exploring and inferring dependencies and relationships of MFs for more understanding;
5. representing the interdependence among MFs of sentences in graph-based structure so as to be more precise and informative;
6. conducting a document linkage process for more effectiveness.

### 3.1. Semantic Text Refining and Annotating Stage

This stage is responsible for refining the text to discover the text MFs with additional annotation for more semantic understanding and aims to

1. accurately parse each sentence and identify POS, subject-action-object, and named-entity recognition;
2. discover the MFs of each sentence in the textual document;
3. exploit semantic information in each sentence through detection attributes of the MFs;
4. reduce the dimensions as much as possible;
5. generate an effective descriptive sentence object (DSO) with a hierarchical sentence object automatically.

This stage can be achieved through these processes. First, the text NL is studied at different linguistic levels, that is, words, sentence, and meaning for semantic analysis and annotation [30, 31]. Second, exploiting the information “who is doing what to whom” clarifies dependencies between verbs and their arguments for extraction of MFs. Finally, building MFs hierarchy-based structure explains sentences MFs and their attributes.

With regard to extracting MFs and building MFs hierarchy-based structure, the following points must be highlighted. First, there is a dire need to refine the text content by representing each sentence with its MFs instead of a series of terms to reduce the dimensions as much as possible. The OpenNLP supports the most common NLP tasks, such as tokenization, sentence segmentation, part-of-speech tagging, named entity extraction, chunking, parsing, and coreference resolution. Furthermore, the AlchemyAPI extracts semantic metadata from content, such as information on subject-action-object relation extraction, people, places, companies, topics, facts, relationships, authors, and languages. Based on the NL concept by OpenNLP and AlchemyAPI, the sentence MFs are identified as subject “Sub,” main verb “MV,” and object “Obj” (direct or indirect object). In addition, such other terms remaining in the sentence as complement “Com” (subject complement or object complement) and modifiers (Mod) are considered as attributes to these MFs.

Second, the automatic annotation is essential for each of the MFs with additional information for more semantic understanding. Based on the semantic annotation by OpenNLP and AlchemyAPI, the sentence MFs are annotated with feature value (FVal), part-of-speech (POS), named-entity recognition (Ner), and a list of feature attributes (FAtts). The list of FAtts is constructed from the remaining terms in the sentence (complement or modifier) relying on the grammatical relation. Each attribute is annotated with attribute value (Val), attribute type (type) which is complement or modifier, attribute POS (POS), and attribute named-entity recognition (Ner).

**Figure 3:**
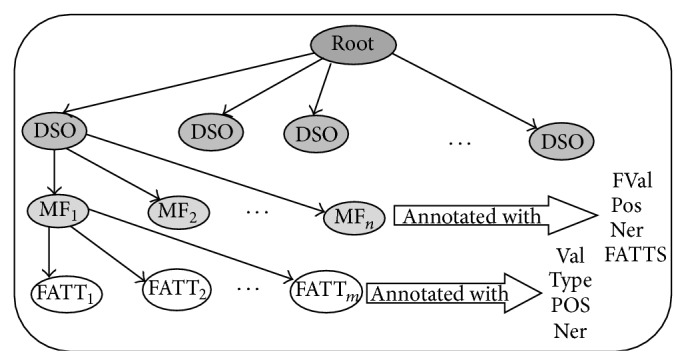
The hierarchy-based structure of the textual document.

Finally, the hierarchy-based structure of the textual document is represented as an object [32] containing the hierarchical structure of sentences with the MFs of the sentences and their attributes. This hierarchical structure maintains the dependency between the terms on the sentence level for more understanding which provides sentences with fast access and retrieval.  Figure 3 shows the hierarchical structure of the text in a theme tree “tree_DSO”: the first level of the hierarchical model denotes all sentence objects that are called DSO, the next level of nodes denotes the MFs of each sentence object, and the last level of nodes denotes the attributes of the MFs.

*tree_DSO.DSO[i]* indicates annotation of sentence object *i*, which is fitted with additional information as in Heuristic 1. This heuristic simplifies the hierarchical model of the textual document with its* DSO* objects and its MFs. The feature score is used to measure the contribution of the MF in the sentence. This score is based on the number of the terms grammatically related to the MF (summation of feature Attributes) as follows:

(1) $$\begin{array}{c} \text{Feature}\;\text{Score}\;\left(\text{FS}\right)=\overset{}{\underset{i}{\sum }}{\text{MF}}_{i}.\text{FAtt}. \end{array}$$

*Heuristic 1*. Let tree_DSO : : = {Sibling  of  DSO  nodes} and let each DSO €  tree_DSO : : = {SEN_key, SEN_value,  SEN_State, Sibling  of  MF_node[]}, where SEN_key indicates the order of the sentence in the document as an index; SEN_value indicates the sentence parsed value as (S(NP(NN employee))(VP(VBZ guides)(NP(DT the)(NN customer)))(·)); SEN_State indicates the sentence tense and state (tense: past; state: negative;); siblings of MF_node[] indicate the list of sibling nodes of MFs for each sentence fitted with Val, POS, Ner, and list of FAtts.

In the text refining and annotating (TRN) algorithm (see Algorithm 1), the textual document is converted to an object model with the hierarchical DSO. The TRN algorithm uses OpenNLP and AlchemyAPI tools for MFs extraction and the hierarchy model construction. As a result, the constructed hierarchy structure leads to the reduction of the dimensions as much as possible and achieves efficient information access and knowledge acquisition.

### 3.2. Dependencies/Relationships Exploring and Inferring Stage

This stage is responsible for exploring how the dependencies and relationships among MFs have an effect and aims to

1. represent the interdependence among sentence MFs in a graph-based structure known as the feature linkage graph (FLG);
2. formulate an accurate measure for the semantic relatedness of MFs;
3. detect the closest synonyms for each of the MFs;
4. infer the relationships and dependencies of MFs with each other.

This stage can be achieved in three processes. First, building an FLG represents the dependencies and relationships among sentences. Second, an efficient MFsSR measure is proposed to contribute to the detection of the closest synonyms and to infer the relationships and dependencies of the MFs. This measure considers the direct relevance and the indirect relevance among MFs. The direct relevance is the synonyms and associated capabilities (similarity, contiguity, contrast, and causality) among the MFs. These association capabilities indicate the relationships that give the largest amount of interdependence among the MFs, while the indirect relevance refers to other relationships among the attributes of these MFs. Finally, the unknown dependencies and relationships among MFs are explored by exploiting semantic information about their texts at a document level.

In addition, a semantic actionable learning agent (SALA) plays a fundamental role in detecting the closest synonyms and inferring the relationships and dependencies of the MFs. Learning is needed to improve the SALA functionality to achieve multiple goals. Many studies show that the RL agent has a high reproductive capability for human-like behaviors [29]. As a result, the SALA performs an adaptive approach combining thesaurus-based and distributional mechanisms. In the thesaurus-based mechanism, words are compared in terms of how they are in the thesaurus (e.g., WordNet), while in the distributional mechanism words are compared in terms of the shared number of contexts in which they may appear.

#### 3.2.1. Graph-Based Organization Constructing Process

The relationships and dependencies among the MFs are organized into a graph-like structure of nodes with links known as FLG and defined in Heuristic 2. The graph structure FLG represents many-to-many relationships among MFs. The FLG is a directed acyclic graph FLG(*V*, *A*) that would be represented in a collection of vertices and a collection of directed arcs. These arcs connect pairs of vertices with no path returning to the same vertex (acyclic). In FLG(*V*, *A*), *V* is the set of vertices or states of the graph, and *A* is the set of arcs between vertices. The FLG construction is based on two sets of data. The first set represents the vertices in a one-dimensional array vertex (*V*) of Feature_Vertex objects (FVOs). The FVO is annotated with Sentence_Feature key (SFKey), feature value (Fval), feature closest synonyms (Fsyn), feature associations (FAss), and feature weight (FW). Where SFKey combines sentence key and feature key in DSO, the sentence key is important because that facilitates accessing the parent of the MFs. The Fval object has the value of the feature, the Fsyn object has a list of the detected closest synonyms of the Fval, and the FAss object has a list of the explored feature associations and relationships for the Fval. In this list, each object has Rel_Typ between two MFs (1 = similarity, 2 = contiguity, 3 = contrast, 4 = causality, and 5 = synonym) and Rel_vertex index of the related vertex. The FW object has accumulated the associations and relationships weights clarifying the importance of the Fval in the textual document. The second set represents the arcs in a two-dimensional array adjacency (*V*, *V*) of Features_link weights (FLW), which indicates the value of the linkage weight in an adjacency matrix between related vertices.

*Heuristic 2*. Let FLG(*V*, *A*):: = 〈Vertex(*V*), Adjacency(*V*, *V*)〉, let Vertex(*V*):: = {FVO_0_, FVO_1_,…, FVO_*n*_}, and let Adjacency(*V*, *V*):: = {{FLW_(0,0)_, FLW_(0,1)_,…}, {FLW_(1,0)_, FLW_(1,1)_,…},…, {…, FLW_(*n*,*n*)_}}, where FVO indicates an object of the MF in vertex vector and annotates with the additional information as FVO : : = {SFKey, Fval, Fsyn, FAss, Fw}; SFKey indicates combination of sentence key and feature key in a DSO; Fval indicates the value of the feature; Fsyn indicates a list of the closest synonyms of the Fval; FAss indicates a list of the feature associations and relationships for the Fval, and each object in this list is defined as FAss.item : : = {Rel_Type, Rel_vertex}, where Rel_Type indicates the value of the relation type linkage between the two features and Rel_vertex indicates the index of related vertex; FW indicates the accumulation of the weights of the associations and relationships of the Fval in the textual document; FLW indicates the value of the linkage weight in an adjacency matrix.

#### 3.2.2. The MFs Semantic Relatedness (MFsSR) Measuring Process

A primary motivation for measuring semantic relatedness comes from the NL processing applications such as information retrieval, information extraction, information filtering, text summary, text annotation, text mining, word sense disambiguation, automatic indexing, machine translation, and other aspects [2, 3]. In this paper, one of the information-based methods is utilized by considering IC. The semantic relatedness that has been investigated concerns the direct relevance and the indirect relevance among MFs. The MFs may be one word or more, and thereby the MF is considered as a concept. The IC is considered as a measure of quantifying the amount of information a concept expresses. Lin [23] assumed that, for a concept *c* proposed IC, let *p*(*c*) be the probability of encountering an instance of concept *c*. The IC value is obtained by considering the negative log likelihood of encountering a concept in a given corpus. Traditionally, the semantic relatedness of concepts is usually based on the cooccurrence information on a large corpus. However, the cooccurrences do not achieve much matching in a corpus, and it is essential to take into account the relationships among concepts. Therefore, development of a new information content method based on the cocontributions instead of the cooccurrences of the proposed MFsSR is important.

The new information content (NIC) measure is an extension of the information content measure. The NIC measures are based on the relations defined in the WordNet ontology. The NIC measure uses hypernym/hyponym, synonym/antonym, holonym/meronymy, and entail/cause to quantify the informativeness of concepts. For example, a hyponym relation could be “bicycle is a vehicle” and a meronym relation could be “a bicycle has wheels.” NIC is defined as a function of the hypernym, hyponym, synonym, antonym, holonym, meronymy, entail, and cause relationships normalized by the maximum number of MFs/attributes objects in the textual document using

(2) NICc=1−log⁡Hype_Hypoc+Syn_Antoc∫     +Holo_Meroc     Hype_Hypoc+Syn_Antoc+Enta_Causec+1    ∫×log⁡⁡(max_concept)−1,

where Hype_Hypo(*c*) returns the number of FVOs in the FLG related to the hypernym or hyponym values, Syn_Anto(*c*) returns the number of FVOs in the FLG related to the synonym or antonym values, Holo_Mero(*c*) returns the number of FVOs in the FLG related to the holonym or meronymy values, and Enta_Cause(*c*) returns the number of FVOs in the FLG related to the entail or cause values. The max_concept is a constant that indicates the total number of MFs/attributes objects in the considered text. The max concept normalizes the NIC value, and hence the NIC values fall in the range of [0, 1].

With the consideration of direct relevance and indirect relevance of MFs, the proposed MFsSR can be stated as follows:

(3) SemRel=λ2∗NICLCSc1,c2NICc1+NICc2+1−λ2∗NICLCSatt_c1,att_c2NICatt_c1+NICatt_c2,

where *λ* ∈ [0, 1] decides the relative contribution of direct and indirect relevance to the semantic relatedness, and because the direct relevance is assumed to be more important than the indirect relevance, *λ* ∈ [0.5, 1].

#### 3.2.3. The Closest-Synonyms Detecting Process

In MFs closest-synonyms detection, the SALA carried out the first action to extract the closest synonyms for each MF, where SALA defies automatic discovery of similar meaning words (synonyms). Due to the important role played by a lexical knowledge base in the closest synonyms detection, SALA adopts the dictionary-based approach to the disambiguation of the MFs. In the dictionary-based approach, the assumption is that the most plausible sense to assign to multiple shared words is that sense that maximizes the relatedness among the chosen senses. In this respect, SALA detects the synonyms by choosing the meaning whose glosses share the largest number of words with the glosses of the neighboring words through lexical ontology. Using a lexical ontology such as WordNet allows the capture of semantic relationships based on the concepts and exploiting hierarchies of concepts besides dictionary glosses. One of the problems that can be faced is that not all synonyms are really related to the context of the document. Therefore, the MFs disambiguation is achieved by implementing the closest-synonym detection (C-SynD) algorithm.

In the C-SynD algorithm, the main task of each SALA is to search for synonyms of each FVO through WordNet. Each synonym and their relationships are assigned in a list called Syn_Relation_List (SRL). Each item in the SRL contains one synonym and its relationships such as* Hypernyms, Hyponym, Meronyms, Holonymy, Antonymy, Entail, *and* Cause*. The purpose of using these relations is to eliminate the ambiguity and polysemy because not all of the synonyms are related to the context of the document. Then, all synonyms and their relationship lists are assigned in a list called F_SRList. The* SALA* starts to filter the irrelevant synonyms according to the score of each SRL. This score is computed based on semantic relatedness between the SRL and every FVO in the Feature_Vertex array as follows:

(4) $$\begin{array}{c} \text{Score}\left({\text{SRL}}_{k}\right)=\overset{m}{\underset{i=0}{\sum }}\;\overset{n}{\underset{j=0}{\sum }}\text{SemRel}\left(\text{SRL}\left(i\right)\text{.item},{\text{FVO}}_{j}\right), \end{array}$$

where *n* is the number of FVOs in the Feature_Vertex array with index *j*, *m* is the number of items in SRL with index *i*, and *k* is index of SRL_*k*_ in F_SRList.

The lists of synonyms and their relationships are used temporarily to serve the C-SynD algorithm (see Algorithm 2). The C-SynD algorithm specifies a threshold value of the score for selecting closest synonyms. SALA applies the threshold to select the closest synonyms with the highest score according to the specific threshold as follows:

(5) $$\begin{array}{c} \text{Closest-Synonyms}=\underset{\text{th}}{\max}\left(\text{F\_SRList}.\text{SRList}.\text{score}\left(\right)\right). \end{array}$$

Then, each SALA retains the closest synonyms in an object Fsyn of each FVO, and SALA links the related FVOs with a bidirectional effect using the SALA_links algorithm (see Algorithm 3). In the SALA_LINKS algorithm, the SALA receives each FVO for detecting links among the other FVOs according to Fsyn object values and FAss object values. The SALA assigns the FLW between two FVOs to their intersection location of the FLG.

#### 3.2.4. The MFs Relationships and Dependencies Inferring Process

In inferring MFs relationships and dependencies, SALA aims to explore the implicit dependencies and relationships in the context such as human relying on four association capabilities. These association capabilities are* similarity, contiguity, contrast (antonym)*,* and causality* [29] which give the largest amount of interdependence among the MFs. These association capabilities are defined as follows.

*Similarity*. For nouns, it is the sibling instance of the same parent instance with is-a relationship in WordNet, indicating hypernym/hyponym relationships. 

*Contiguity*. For nouns, it is the sibling instance of the same parent instance with part-of relationship in WordNet, indicating holonym/meronymy relationship. 

*Contrast*. For adjectives or adverbs, it is the sibling instance of the same parent instance with is-a relationship and with antonym (opposite) attribute in WordNet, indicating a synonym/antonym relationship. 

*Causality*. For verbs, it indicates the connection of the sequence of events by a cause/entail relationship, where a cause picks out two verbs: one of the verbs is causative such as (give) and the other is called resultant such as (have) in WordNet.

To equip SALA with decision-making and experience learning capabilities to achieve multiple-goal RL, this study utilizes the RL method relying on *Q*-learning to design a SALA inference engine with sequential decision-making. The objective of* SALA* is to select an optimal association to maximize the total long-run accumulated reward. Hence, SALA can achieve the largest amount of interdependence among MFs through implementing the inference optimal association capabilities with the *Q*-learning (IOAC-QL) algorithm. 

(*1) Utilizing Multiple-Goal Reinforcement Learning (RL)*. RL specifies what to do but not how to do it through the reward function. In sequential decision-making tasks, an agent needs to perform a sequence of actions to reach goal states or multiple-goal states. One popular algorithm for dealing with sequential decision-making tasks is *Q*-learning [33]. In a *Q*-learning model, a *Q*-value is an evaluation of the “quality” of an action in a given state. *Q*(*x*, *a*) indicates how desirable action *a* is in the state *x*. SALA can choose an action based on *Q*-values. The easiest way of choosing an action is to choose the one that maximizes the *Q*-value in the current state.

To acquire the *Q*-values, the algorithm *Q*-learning is used to estimate the maximum discounted cumulative reinforcement that the model will receive from the current state *x* on max⁡(∑_*i*=0_ *γ* ^*i*^ *r*_*i*_), where *γ* is a discount factor and *r*_*i*_ is the reward received at step *i* (which may be 0). The updating of *Q*(*x*, *a*) is based on minimizing *r* + *γf*(*y*) − *Q*(*x*, *a*), *f*(*y*) = max⁡_*a*_ *Q*(*y*, *a*), and *y* is the new state resulting from action *a*. Thus, updating is based on the temporal difference in evaluating the current state and the action chosen. Through successive updates of the *Q* function, the model can learn to take into account future steps in longer and longer sequences, notably without explicit planning [33]. SALA may eventually converge to a stable function or find an optimal sequence that maximizes the reward received. Hence, the SALA learns to address sequential decision-making tasks.

*Problem Formulation*. The *Q*-learning algorithm is used to determine the best possible action, that is, selecting the most effective relations, analyzing the reward function, and updating the related feature weights accordingly. The promising action can be verified by measuring the relatedness score of the action value with the remained MFs using a reward function. The formulation of the effective association capabilities exploration is performed by estimating an action-value function. The value of taking action *a* in state *s* is defined under a policy *π*, denoted by *Q*(*s*; *a*), as the expected return starting from *s* and taking the action *a*. Action policy *π* is a description of the behavior of the learning SALA. The policy is the most important criterion in providing the RL with the ability to determine the behavior of the SALA [29].

In single goal reinforcement learning, these *Q*-values are used only to rank order of the actions in a given state. The key observation here is that the *Q*-values can also be used in multiple-goal problems to indicate the degree of preference for different actions. The available way in this paper to select a promising action to execute is to generate an overall *Q*-value as a simple sum of the *Q*-values of the individual SALA. The action with the maximum summed value is then chosen to execute. 

*Environment, State, and Actions*. For the effective association capabilities exploration problem, the FLG is provided as an environment in which intelligent agents can learn and share their knowledge and can explore dependencies and relationships among MFs. The SALAs receive the environmental information data as a state and execute the elected action in the form of an action value table, which provides the best way of sharing knowledge among agents. The state is defined by the status of the MF space. In each state, the MF object is fitted with the closest-synonyms relations; there is a set of actions. In each round, the SALA decides to explore the effective associations that gain the largest amount of interdependence among the MFs. Actions are defined by selecting a promising action with the highest reward, by learning which action is the optimal for each state. In summary, once *s*_*k*_ (MF object) is received from the FLG, SALA chooses one of the associations as the action *a*_*k*_ according to SALA's policy *π* ^*^(*s*_*k*_), executes the associated action, and finally returns the effective relations. Heuristic 3 defines the action variable and the optimal policy.

*Heuristic 3 (action variable)*. Let *a*_*k*_ symbolize the action that is executed by SALA at round *k*:

(6) $$\begin{array}{c} a_{k}=\pi^{*}\left(s_{k}\right). \end{array}$$

Therein, one has the following:

- *a*_*k*_  € action space {similarity; contiguity; contrast; and  causality};
- *π* ^*^(*s*_*k*_) = argmax⁡_*a*_ *Q* ^*^(*s*_*k*_, *a*_*k*_).

Once the optimal policy (*π* ^*^) is obtained, the agent chooses the actions using the maximum reward. 

*Reward Function*. The reward function in this paper measures the dependency and the relatedness among MFs. The learner is not told which actions to take as in most forms of machine learning. Rather, the learner must discover which actions yield the most reward by trying them [33]. 

(*2) The SALA Optimal Association/Relationships Inferences*. The optimal association actions are achieved according to the IOAC-QL algorithm. This algorithm starts to select the action with the highest expected future reward from each state. The immediate reward, which the SALA gets to execute an action from a state** s**, plus the value of an optimal policy, is the *Q*-value. The higher *Q*-value points to the greater chance of that action being chosen. First, initialize all the *Q*(*s*, *a*) values to zero. Then, each SALA performs the following steps in Heuristic 4. 

*Heuristic 4*

(i) At every round *k*, select one action *a*_*k*_ from all the possible actions: (similarity, contiguity, contrast, and causality), and execute it.
(ii) Receive the immediate reward *r*_*k*_ and observe the new state *s*_*k*_′.
(iii) Get the maximum *Q*-value of the state *s*_*k*_′ based on all the possible actions *a*_*k*_ = *π* ^*^(*s*_*k*_) = argmax⁡_*a*_ *Q* ^*^(*s*_*k*_, *a*_*k*_).
(iv) Update the *Q*(*s*_*k*_, *a*_*k*_). as follows:
  (7) Qsk,ak=1−αQsk,ak+αrsk,ak+γmax⁡a′∈Ask⁡Qsk′,ak′,
 where *Q*(*s*_*k*_, *a*_*k*_) is worthy of selecting the action (*a*_*k*_) at the state (*s*_*k*_). *Q*(*s*_*k*_′, *a*_*k*_′) is worthy of selecting the next action (*a*_*k*_′) at the next state (*s*_*k*_′). The *r*(*s*_*k*_, *a*_*k*_) is a reward corresponding to the acquired payoff. A (*s*_*k*_′) is a set of possible actions at the next state (*s*_*k*_′). *α* (0 < *α* ≤ 1) is learning rate. *γ* (0 ≤ *γ* ≤ 1) is discount rate.

During each round after taking the action *a*, the action value is extracted from WordNet. The weight of the action value is measured to represent the immediate reward, where the SALA computes the weight AVW_*i*,*k*_ for the action value *i* in document *k* as follows:

(8) Rewardr=AVWi,k= AVWi,k+∑j=1nFWj,k∗⁡SemRel(i,j),

where *r* is the reward of the selected action *a*_*i*_, AVW_*i*,*k*_ is the weight of action_value *i* in document *k*, FW_*j*,*k*_ is the weight of each feature object FVO_*j*_ in document *k*, and SemRel(*i*, *j*) is the semantic relatedness between the action_value *i* and feature object FVO_*j*_.

The most popular weighting scheme is the normalized word frequency TFIDF [34], used to measure AVW_*i*,*k*_ and FW_*j*,*k*_. The FW_*j*,*k*_ in (8) is calculated likewise AVW_*i*,*k*_ is based on (9) and (10).

*Action_value weight (AVW*_*i*,*k*_) is a measure to calculate the weight of the action_value that is the scalar product of action_value frequency and inverse document frequency as in (9). The action_value frequency (*F*) measures the importance of this value in a document. Inverse document frequency (IDF) measures the general importance of this action_value in a corpus of documents:

(9) $$\begin{array}{c} {\text{AVW}}_{i,k}=\frac{F_{ik}}{\sum_{k}F_{i,k}}*{\text{IDF}}_{i}, \end{array}$$

where *F*_*i*,*k*_ represents the number of this action_value *i* cocontributions in document *d*_*k*_, normalized by the number of cocontributions of all MFs in document *d*_*k*_, normalized to prevent a bias towards longer documents. IDF is performed by dividing the number of all documents by the number of documents containing this action_value defined as follows:

(10) $$\begin{array}{c} {\text{IDF}}_{i}=\log\left(\frac{\left|D\right|}{\left|\left\{d_{k}:v_{i}\in d_{k}\right\}\right|}\right), \end{array}$$

where |*D*| is the total number of documents in the corpus and |{*d*_*k*_ : *v*_*i*_ ∈ *d*_*k*_}| is the number of documents containing action_value *V*_*i*_.

**Figure 4:**
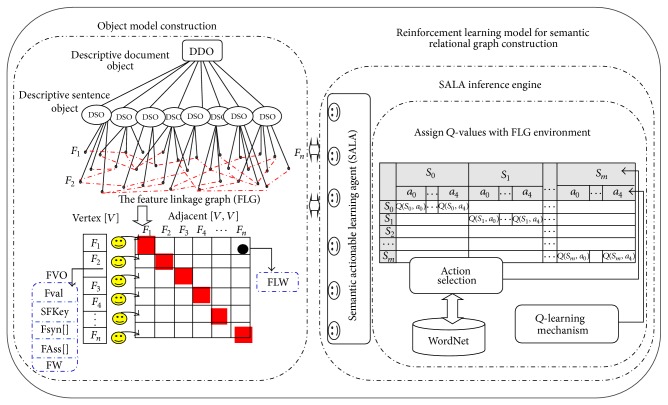
The IOAC-QL algorithm implementation.

Thus, in the IOAC-QL algorithm (see Algorithm 4) implementation shown in Figure 4, the SALA selects the optimal action with the higher reward. Hence, each SALA retains effective actions in the FAss list object of each FVO. In this list, each object has Rel-Type object and Rel_vertex object. The Rel-Type object value equals the action_type object value, and Rel_vertex object value equals the related vertex index. The SALA then links the related FVOs with the bidirectional effect using the* SALA_links* algorithm and sets the FLW value of the linkage weight in the adjacency matrix with the reward of the selected action.

An example of monitoring the SALA decision through the practical implementation of the IOAC-QL algorithm actions is illustrated in Figure 4.

For state FVO_1_, one has the following:

 action similarity: ActionResult State FVO_1_ FVO_7_ FVO_9_ FVO_22_ FVO_75_ FVO_92_ FVO_112_ FVO_125_, Prob. 8, reward 184, PrevState FVO_1_, *Q*(*s*, *a*) = 91;
 action contiguity: ActionResult State FVO_103_ FVO_17_ FVO_44_, Prob. 3, reward 74, PrevState FVO_1_, *Q*(*s*, *a*) = 61;
 action contrast: ActionResult State FVO_89_, Prob. 1, reward 22, PrevState FVO_1_, *Q*(*s*, *a*) = 32.9;
 action causality: ActionResult State FVO_63_ FVO_249_, Prob. 2, reward 25, PrevState FVO_1_, *Q*(*s*, *a*) = 42.9.

As revealed in the case FVO_1_, the SALA receives the environmental information data FVO_1_ as a state, and then the SALA starts the implementation to select the action with the highest expected future reward among the other FVOs. The result of each action is illustrated, the action with the highest reward is selected, and the elected action is executed. The SALA decision is accomplished in FLG with the effective extracted relationships according to *Q*(*s*, *a*) value as shown in Table 1.

### 3.3. Document Linkage Conducting Stage

This section presents an efficient linkage process at the corpus level for many documents. Due to the SHGRS scheme of each document, measuring the commonality between the FLG of the documents through finding all distinct common similarity or relatedness subgraphs is required. The objective of finding common similar or related subgraphs as a relatedness measure is to be utilized in document mining and managing processes such as clustering, classification, and retrieval. It is essential to establish that the measure is metric. A metric measure ascertains the order of objects in a set, and hence operations such as comparing, searching, and indexing of the objects can be performed.

Graph relatedness [35, 36] involves determining the degree of similarity between these two graphs (a number between 0 and 1), so the same node in both graphs would be similar if its related nodes were similar. The selection of subgraphs for matching depends on detecting the more important nodes in FLG using FVO.Fw value. The vertex with the highest weight is the first node selected, which then detects all related vertices from an adjacency matrix as a subgraph. This process depends on computing the maximum common subgraphs. The inclusion of all common relatedness subgraphs is accomplished through recursive iterations and holding onto the value of the relatedness index of the matched subgraphs. Eliminating the overlap is performed using a dynamic programming technique that keeps track of the nodes visited and their attribute relatedness values.

The function relatedness subgraphs utilize an array Rel-Nodes[] of the related vertices for each document. The function SimRel(*x*_*i*_, *y*_*i*_) computes the similarity between the two nodes *x*_*i*_, *y*_*i*_ as defined in (11). The subgraph matching process is measured using the following formula:

(11) Sub-graph-RelSG1,SG2 =∑iSG1∑jSG2SemRelNi,Nj∑iSG1Ni+∑jSG2Nj,

where SG1 is the selected subgraph from the FLG of one document, SG2 is the selected subgraph from the FLG of another document, and SemRel(*N*_*i*_, *N*_*j*_) is the degree of matching for both nodes *N*_*i*_ and *N*_*j*_. A threshold value for node matching is also utilized; the threshold is set to a 50% match.

## 4. Evaluation of the Experiments and Results

This evaluation is especially vital, as the aim of building the SHGRS is to use them efficiently and effectively for the further mining process. To explore the effectiveness of the proposed SHGRS scheme, examining the correlation of the proposed semantic relatedness measure compared with other previous relatedness measures is required. The impact of the proposed MFsSR for detecting the closest synonym is studied and compared to the PMI [25, 37] and independent component analysis (ICA) [38] for detecting the best near-synonym. The difference between the proposed discovering semantic relationships or associations IOAC-QL, implicit relation extraction conditional random fields (CRFs) [6, 39], and term frequency and inverse cluster frequency (TFICF) [40] is computed.

A case study of a managing and mining task (i.e., semantic clustering of text documents) is considered. This study shows how the proposed approach is workable and how it can be applied to mining and managing tasks. Experimentally, significant improvements in all performances are achieved in the document clustering process. This approach outperforms conventional word-based [16] and concept-based [17, 41] clustering methods. Moreover, performance tends to improve as more semantic analysis is achieved in the representation.

### 4.1. Evaluation Measures

Evaluation measures are subcategorized into text quality-based evaluation, content-based evaluation, coselection-based evaluation, and task-based evaluations. The first category of evaluation measures is based on text quality using aspects such as grammaticality, nonredundancy, referential clarity, and coherence. For content-based evaluations, measures such as similarity, semantic relatedness, longest common subsequence, and other scores are used. The third category is based on coselection evaluation using precision, recall, and *F*-measure values. The last category, task-based evaluation, is based on the performance of accomplishing the given task. Some of these tasks are information retrieval, question answering, document classification, document summarizing, and document clustering methods. In this paper, the content-based, the coselection-based, and the task-based evaluations are used to validate the implementation of the proposed scheme over the real corpus of documents.

#### 4.1.1. Measure for Content-Based Evaluation

The relatedness or similarity measures are inherited from probability theory and known as the correlation coefficient [42]. The correlation coefficient is one of the most widely used measures to describe the relatedness *r* between two vectors, *X* and *Y*.

*Correlation Coefficient r*. The correlation coefficient is a relatively efficient relatedness measure, which is a symmetrical measure of the linear dependence between two random variables.

Therefore, the correlation coefficient can be considered as the coefficient for the linear relationship between corresponding values of *X* and *Y*. The correlation coefficient *r* between sequences *X* = {*x*_*i*_ : *i* = 1,…, *n*} and *Y* = {*y*_*i*_ : *i* = 1,…, *n*} is defined by

(12) $$\begin{array}{c} r=\frac{\sum_{i=1}^{n}X_{i}Y_{i}}{\sqrt{\left(\sum_{i=1}^{n}X_{i}^{2}\right)*\left(\sum_{i=1}^{n}Y_{i}^{2}\right)}}. \end{array}$$

*Acceptance Rate (AR)*. Acceptance rate is a proportion of correctly predicted similar or related sentences compared to all related sentences. High acceptance rate means recognizing almost all similar or related sentences:

(13) $$\begin{array}{c} \text{AR}=\frac{\text{TP}}{\left(\text{TP}+\text{FN}\right)}. \end{array}$$

*Accuracy (Acc)*. Accuracy is a proportion of all correctly predicted sentences compared to all sentences:

(14) $$\begin{array}{c} \text{Acc}=\frac{\text{TP}+\text{TN}}{\left(\text{TP}+\text{FN}+\text{FP}+\text{TN}\right)}, \end{array}$$

where TP, TN, FP, and FN stand for true positive (the number of pairs correctly labeled as similar), true negative (the number of pairs correctly labeled as dissimilar), false positive (the number of pairs incorrectly labeled as similar), and false negative (the number of pairs incorrectly labeled as dissimilar) as shown in Table 2.

#### 4.1.2. Measure for Coselection-Based Evaluation

For all the domains, a precision *P*, recall *R*, and *F*-measure are utilized as the measures of performance in the coselection-based evaluation. These measures may be defined via computing the correlation between the extracted, correct, and wrong closest synonyms or semantic relationships/dependencies. Let TP denote the number of correctly detected closest synonyms or semantic relationships explored, let FP be the number of incorrectly detected closest synonyms or semantic relationships explored, and let FN be the number of correctly but not detected closest synonyms or semantic relationships explored in a dataset. The *F*-measure combines the precision and recall in one metric and is often used to show the efficiency. This measurement can be interpreted as a weighted average of the precision and recall, where an *F*-measure reaches its best value at 1 and worst value at 0. Precision, recall, and *F*-measure are defined as follows:

(15) $$\begin{array}{c} \text{Recall}=\frac{\text{TP}}{\left(\text{TP}+\text{FN}\right)},\\ \text{Precision}=\frac{\text{TP}}{\left(\text{TP}+\text{FP}\right)},\\ F\text{-measure}=\frac{2\left(\text{Recall}*\text{Precision}\right)}{\left(\text{Recall}+\text{Precision}\right)}. \end{array}$$

#### 4.1.3. Measure for Task-Based Evaluation

A case study of a managing and mining task (i.e., semantic clustering of text documents) is studied for task-based evaluation. The test is performed with the standard clustering algorithms that accept the pairwise (dis)similarity between documents rather than the internal feature space of the documents. The clustering algorithms may be classified as listed below [43]: (a) flat clustering (which creates a set of clusters without any explicit structure that would relate the clusters to each other, also called exclusive clustering), (b) hierarchical clustering (which creates a hierarchy of clusters), (c) hard clustering (which assigns each document/object as a member of exactly one cluster), and (d) soft clustering (which distributes the documents/objects over all clusters). These algorithms are agglomerative (hierarchical clustering), *K*-means (flat clustering, hard clustering), and EM algorithm (flat clustering, soft clustering).

Hierarchical agglomerative clustering (HAC) and the *K*-means algorithm have been applied to text clustering in a straightforward way [16, 17]. The algorithms employed in the experiment included *K*-means clustering and HAC. To evaluate the quality of the clustering, the *F*-measure is used for quality measures, which combine the precision and recall measures. These metrics are computed for every (class, cluster) pair. The precision *P* and recall *R* of a cluster *S*_*i*_ with respect to a class *L*_*r*_ are defined as

(16) $$\begin{array}{c} P\left(L_{r},S_{i}\right)=\frac{N_{ri}}{N_{i}},\\ R\left(L_{r},S_{i}\right)=\frac{N_{ri}}{N_{r}}, \end{array}$$

where *N*_*ri*_ is the number of documents of class *L*_*r*_ in cluster *S*_*i*_, *N*_*i*_ is the number of documents of cluster *S*_*i*_, and *N*_*r*_ is the number of documents of class *L*_*r*_.

The *F*-measure, which is a harmonic mean of precision and recall, is defined as

(17) $$\begin{array}{c} F\left(L_{r},S_{i}\right)=\frac{2*P\left(L_{r},S_{i}\right)*R\left(L_{r},S_{i}\right)}{P\left(L_{r},S_{i}\right)+R\left(L_{r},S_{i}\right)}. \end{array}$$

A per-class *F* score is calculated as follows:

(18) $$\begin{array}{c} F\left(L_{r}\right)=\underset{S_{i}\in C}{\max}\;F\left(L_{r},S_{i}\right). \end{array}$$

With respect to class *L*_*r*_, the cluster with the highest *F*-measure is considered to be the cluster that maps to class *L*_*r*_ that *F*-measure becomes the score for class *L*_*r*_, and *C* is total number of clusters. The overall *F*-measure for the clustering result *C* is the weighted average of the *F*-measure for each class *L*_*r*_ (macroaverage):

(19) $$\begin{array}{c} FC=\frac{\sum_{L_{r}}^{}\left(\left|N_{r}\right|*F\left(L_{r}\right)\right)}{\sum_{L_{r}}^{}\left|N_{r}\right|}, \end{array}$$

where |*N*_*r*_| is the number of objects in class *L*_*r*_. The higher the overall *F*-measure is, the better the clustering is due to the higher accuracy of the clusters mapping to the original classes.

### 4.2. Evaluation Setup (Dataset)

Content-based, coselection-based, and task-based evaluations are used to validate experimenting over the real corpus of documents; the results are very promising. The experimental setup consists of some datasets of textual documents as detailed in Table 3. Experiment  1 uses the Miller and Charles (MC) and Microsoft Research Paraphrase Corpus. Experiment  2 uses the British National Corpus, TREC, IJCNLP 2011 (NYT and Wikipedia), SN, and BLESS. Experiment  3 uses The Reuters-21,578 and 20 newsgroups.

### 4.3. Evaluation Results

This section reports on the results of three experiments conducted using the evaluation datasets outlined in the previous section. The SHGRS is implemented and evaluated based on concept analysis and annotation as sentence based in experiment  1, document based in experiment  2, and corpus based in experiment  3. In experiment  1, the DS1 and DS2 investigated the effectiveness of the proposed MFsSR compared to previous studies based on the availability of semantic relatedness values of human judgments and previous studies. In experiment  2, the DS3 and DS4 investigated the effectiveness of the closest synonym detection using MFsSR compared to using PMI [25, 37] and ICA [38] based on the availability of synonyms coming from three sources: WordNet 3.0, Roget's thesaurus, and a synonyms database. Furthermore, the DS5, DS6, DS7, and DS8 investigated the effectiveness of the IOAC-QL for semantic relationships exploration compared to the implicit relation extraction CRFs [6, 39] and TFICF [40] based on the availability of semantic relations judgments. In experiment  3, the DS9 and DS10 investigated the effectiveness of the document linkage process for clustering case study than previous studies as word-based [16] and concept-based [17] based on the availability of human judgments.

These experiments revealed some interesting trends in terms of text organization and a representation scheme based on semantic annotation and reinforcement learning. The results show the effectiveness of the proposed scheme for accurate understanding of the underlying meaning. The proposed SHGRS is exploited in mining and managing operations such as gathering, filtering, searching, retrieving, extracting, classifying, clustering, and summarizing documents. In addition, the experiments illustrate the improvements when direct relevance and indirect relevance are included in the process of measuring semantic relatedness between MFs of documents. The main purpose of semantic relatedness measures is to infer unknown dependencies and relationships among the MFs. The proposed MFsSR is a backbone of the closest synonym detection and semantic relationships exploration algorithms. The performance of MFsSR for closest synonym detection and IOAC-QL for semantic relationships exploration algorithms is proportionally improved as well as having a semantic understanding of text content achieved. The relatedness subgraph measure is used for the document linkage process at the corpus level. Through this process, better clustering results can be achieved as more attempt to improve the accuracy of measuring the relatedness between documents using semantic information in SHGRS. The relatedness subgraphs measure affects the performance of the output of the document managing and mining process relative to how much of the semantic information it considers. The most accurate relatedness can be achieved when the maximum amount of the semantic information is provided. Consequently, the quality of clustering is improved, and when semantic information is considered, the approach surpasses the word-based and concept-based techniques.

#### 4.3.1. Experiment  1: Comparative Study (Content-Based Evaluation)

This experiment shows the necessity to evaluate the performance of the proposed MFsSR based on a benchmark dataset of human judgments, so the results of the proposed semantic relatedness would be comparable with other previous studies in the same field. In this experiment, they attempted to compute the correlation between the ratings of the proposed semantic relatedness approach and the mean ratings reported by Miller and Charles (DS1 in Table 3). Furthermore, the results produced are compared against eight other semantic similarities approaches, namely, Rada, Wu and Palmer, Resnik, Jiang and Conrath, Lin, T. Hong and D. Smith, and Zili Zhou [21].

Table 4 shows the correlation coefficient results between nine componential approaches and the Miller and Charles ratings mean. The semantic relatedness for the proposed approach outperformed all the listed approaches. Unlike all the listed methods, in the proposed semantic relatedness, different properties are considered. Furthermore, the good correlation value of the approach also results from considering all available relationships between concepts and the indirect relationships between attributes of each concept when measuring the semantic relatedness. In this experiment, the proposed approach achieved a good correlation value with the human-subject rating reported by Miller and Charles. Based on the results of this study, the MFsSR correlation value has proven that considering the direct/indirect relevance is specifically important for at least 6% improvement over the best previous results. This improvement contributes to the achievement of the largest amount of relationships and interdependence among MFs and their attributes at the document level and to the document linkage process at a corpus level.

Table 5 summarizes the characteristics of the MRPC dataset (DS2 in Table 3) and presents comparison of the Acc and AR values between the proposed semantic relatedness measure MFsSR and Islam and Inkpen [7]. Different relatedness thresholds ranging from 0 to 1 with interval 0.1 are used to validate the MFsSR with Islam and Inkpen [7]. After evaluation, the best relatedness thresholds of Acc and AR are 0.6, 0.7, and 0.8. These results indicate that the proposed MFsSR surpasses Islam and Inkpen [7] in terms of Acc and AR. The results of each approach listed below were based on the best Acc and AR through all thresholds instead of under the same relatedness threshold. This improvement in Acc and AR values is due to the increase in the numbers of pairs predicted correctly after considering direct/indirect relevance. This relevance takes into account the closest synonym and the relationships of sentences MFs.

#### 4.3.2. Experiment  2: Comparative Study of Closest-Synonym Detection and Semantic Relationships Exploration

This experiment shows the necessity to study the impact of the MFsSR for inferring unknown dependencies and relationships among the MFs. This impact is achieved through the closest-synonym detection and semantic relationship exploration algorithms, so the results would be comparable with other previous studies in the same field. Throughout this experiment, the impact of the proposed MFsSR for detecting the closest synonym is studied and compared to the PMI approach and ICA approach for detecting the best near-synonym. The difference among the proposed discovering semantic relationships or associations IOAC-QL, the implicit relation extraction CRFs approach, and TFICF approach is examined. This experiment attempts to measure the performance of the MFsSR for the closest synonym detection and the IOAC-QL for semantic relationship exploration algorithms. Hence, more semantic understanding of the text content is achieved by inferring unknown dependencies and relationships among the MFs. Considering the direct relevance among the MFs and the indirect relevance among the attributes of the MFs in the proposed MFsSR constitutes a certain advantage over previous measures. However, most of the time, the incorrect items are due to a wrong syntactic parsing from the OpenNLP parser and AlchemyAPI. According to the preliminary study, it is certain that the accuracy of parsing tools' effects is on the performance of the MFsSR.

Detecting the closest synonym is a process of MF disambiguation resulting from the implementation of the MFsSR technique and the threshold of the synonyms scores through the C-SynD algorithm. In the C-SynD algorithm, the MFsSR takes into account the direct relevance among MFs and the indirect relevance among MFs and their attributes in a context that gives a significant increase in the recall without disturbing the precision. Thus, the MFsSR between two concepts considers the information content with most of WordNet relationships.  Table 6 illustrates the performance of the C-SynD algorithm based on the MFsSR compared to the best near-synonym algorithm using the PMI and ICA approaches. This comparison was conducted for two corpora of text documents that are BNC and NS (DS3, and DS4 in Table 3). As indicated in Table 6, the C-SynD algorithm yielded higher average precision, recall, and *F*-measure values than PMI approach by 25%, 20%, and 21% on SN dataset, respectively, and also by 8%, 4%, and 7% on BNC dataset. Furthermore, the C-SynD algorithm also yielded higher average precision, recall, and *F*-measure values than ICA approach by 6%, 9%, and 7% on SN dataset, respectively, and also by 6%, 4%, and 5% on BNC dataset. The improvements achieved in the performance values are due to the increase of the number of pairs predicted correctly and the decrease of the number of pairs predicted incorrectly through implementing MFsSR. The important observation from this table is the improvements achieved in recall which measures effectiveness of the C-SynD algorithm. Achieving better precision values is clear, with a high percentage in different datasets and domains.

Inferring the MFs relationships and dependencies is a process of achieving a large number of interdependences among MFs resulting from the implementation of the IOAC-QL algorithm. In the IOAC-QL algorithm, the *Q*-learning is used to select the optimal action (relationships) that gains the largest amount of interdependences among the MFs resulting from the measure of the AVW.  Table 7 illustrates the performance of the IOAC-QL algorithm based on AVWs compared to the implicit relationship extraction based on CRFs and TFICF approaches, which was conducted over the TREC, Bless, the NYT, and the Wikipedia corpus (DS5, DS6, DS7, and DS8 in Table 3). The data in this table shows increases in precision, recall, and *F*-measure values due to the increase in the number of pairs predicted correctly after considering direct/indirect relevance through the expansion of closest synonym. In addition, the IOAC-QL algorithm, the CRFs, and the TFICF approaches are beneficial to the extraction performance, but the IOAC-QL contributes more than CRFs and TFICF. Thus, IOAC-QL considers similarity, contiguity, contrast, and causality relationships between MFs and its closest-synonyms, while the CRFs and TFICF consider is-a and part-of relationships only between concepts. The improvements of the *F*-measure were achieved through an IOAC-QL algorithm up to 23% for TREC dataset, 22% for BLESS dataset, 21% for NYT dataset, and 6% for Wikipedia dataset, approximately from the CRFs approach. Furthermore, the IOAC-QL algorithm also yielded higher *F*-measure values than TFICF approach by 10% for TREC dataset, 17% for BLESS dataset, and 7% for NYT dataset, respectively.

#### 4.3.3. Experiment  3: Comparative Study (Task-Based Evaluation)

This experiment shows the effectiveness of the proposed semantic representation scheme in document mining and managing operations such as gathering, filtering, searching, retrieving, extracting, classifying, clustering, and summarizing documents. Many sets of experiments using the proposed scheme on different datasets in text clustering have been conducted. The experiments demonstrate the extensive comparisons between the MF-based analysis and the traditional analysis. Experimental results demonstrate the substantial enhancement of the document clustering quality. In this experiment, the application of the semantic understanding of the textual documents is presented. Textual documents are parsed to produce the rich syntactic and semantic representations of SHGRS. Based on these semantic representations, document linkages are estimated using the subgraph matching process that is based on finding all distinct similarity common subgraphs. The semantic representations of textual documents determining the relatedness between them are evaluated by allowing clustering algorithms to use the produced relatedness matrix of the document set. To test the effectiveness of subgraph matching in determining an accurate measure of the relatedness between documents, an experiment using the MFs-based analysis and subgraphs relatedness measures was conducted. Two standard document clustering techniques were chosen for testing the effect of the document linkages on clustering [43]: (1) HAC and (2) *K*-means. The MF-based weighting is the main factor that captures the importance of a concept in a document. Furthermore, document linkages using the subgraph relatedness measure are among the main factors that affect clustering techniques. Thus, to study the effect of the MFs-based weighting and the subgraph relatedness measure (graph-based approach) on the clustering techniques, this experiment is repeated for the different clustering techniques as shown in Table 8. Basically, the aim is to maximize the weighted average of the *F*-measure for each class (FC) of clusters to achieve high-quality clustering.

Table 8 shows that the graph-based approach achieves higher performance than term-based and concept-based approaches. This comparison was conducted for two corpora of text documents that are the Reuters-21578 dataset and the 20 newsgroups dataset (DS9 and DS10 in Table 3). The results shown in Table 8 illustrate improvement in the clustering quality when more attributes are included in graph-based relatedness measure. The improvements were achieved at the graph-based approach up to 17% by HAC and 40% by *K*-means for Reuters-21578 dataset while up to 36% by HAC and 39% by *K*-means for approximately 20 newsgroup datasets from the base case (term-based) approach. Furthermore, the improvements also were achieved at the graph-based approach up to 5% by HAC and 7% by *K*-Means for Reuters-21578 dataset while up to 7% by HAC and 8% by *K*-Means for approximately 20 Newsgroup datasets from Concept-based approach.

## 5. Conclusions

This paper proposed the SHGRS to improve the effectiveness of the mining and managing operations in textual documents. Specifically, a three-stage approach was proposed for constructing the scheme. First, the MFs with their attributes are extracted, and hierarchy-based structure is built to represent sentences by the MFs and their attributes. Second, a novel semantic relatedness computing method was proposed for inferring relationships and dependencies of MFs, and the relationship between MFs is represented by a graph-based structure. Third, a subgraph matching algorithm was conducted for document linkage process. The documents clustering is used as a case study and conducted extensive experiments on 10 different datasets, and the results showed that the scheme can result in a better mining performance compared with traditional approach. Future work will focus on conducting other case studies to processes such as gathering, filtering, retrieving, classifying, and summarizing information.

## Figures and Tables

**Algorithm 1:**
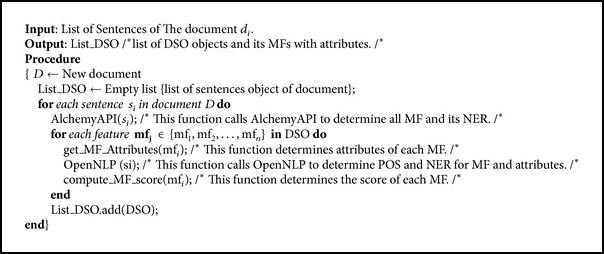
TRN algorithm.

**Algorithm 2:**
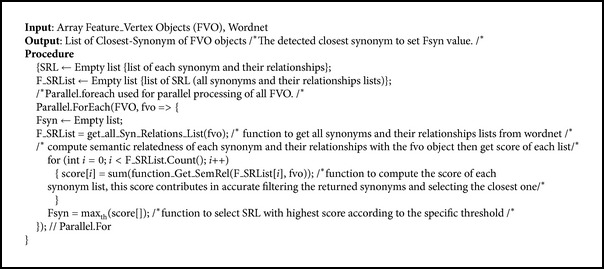
C-SynD algorithm.

**Algorithm 3:**
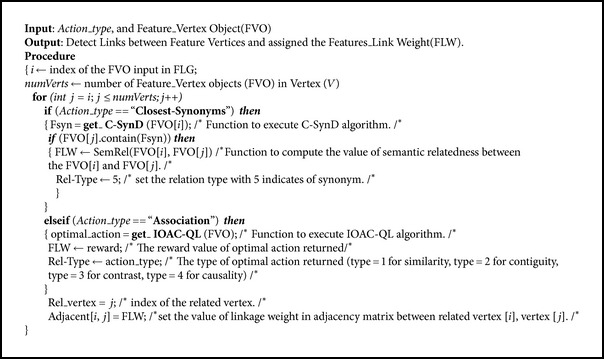
SALA_links algorithm.

**Algorithm 4:**
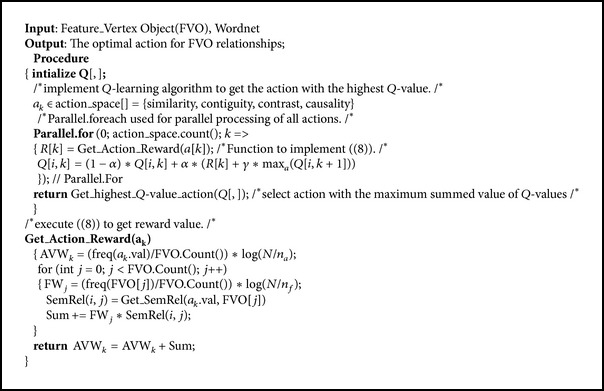
IOAC-QL algorithm.

**Table 1:** Action values table with the SALA decision.

State	FVO_1_	FVO_1_	FVO_1_	FVO_1_
Action	Similarity	Contiguity	Contrast	Causality
ActionResult State	FVO_1_ FVO_7_ FVO_9_ FVO_22_ FVO_75_ FVO_92_ FVO_112_ FVO_125_	FVO_103_ FVO_17_ FVO_44_	FVO_89_	FVO_63_ FVO_249_
Total reward	184	74	22	25
*Q*(*s*, *a*)	94	61	32.9	42.9
Decision	Yes	No	No	No

**Table 2:** Contingency table presents human judgment and system judgment.

	Human judgment	
	True	False
System judgment		
True	TP	FP
False	FN	TN

**Table 3:** Datasets details for the experimental setup.

Dataset	Experimenting	Dataset Name	Description
DS1	Experiment 1: content-based evaluation	Miller and Charles (MC)^1^	RG consists of 65 pairs of nouns extracted from the WordNet, rated by multiple human annotators.
DS2		Microsoft Research Paraphrase Corpus (MRPC)^2^	The corpus consists of 5,801 sentence pairs collected from newswire articles, 3,900 of which were labeled as relatedness by human annotators. The whole set is divided into a training subset (4,076 sentences of which 2,753 are true) and a test subset (1,725 pairs of which 1,147 are true).

DS3	Experiment 2: coselection-based evaluation (closest-synonym detection)	British National Corpus (BNC)^3^	BNC is a carefully selected collection of 4124 contemporary written and spoken English texts, contains 100-million-word text corpus of samples of written and spoken English with the near-synonym collocations.
DS4		SN (semantic neighbors)^4^	SN relates 462 target terms (nouns) to 5910 relatum terms with 14.682 semantic relations (7341 are meaningful and 7341 are random). The SN contains synonyms coming from three sources: WordNet 3.0, Roget's thesaurus, and a synonyms database.

DS5	Experiment 2: coselection-based evaluation (semantic relationships exploration)	BLESS^6^	BLESS relates 200 target terms (100 animate and 100 inanimate nouns) to 8625 relatum terms with 26.554 semantic relations (14.440 are meaningful (correct) and 12.154 are random). Every relation has one of the following types: hypernymy, cohypernymy, meronymy, attribute, event, or random.
DS6		TREC^5^	TREC includes 1437 sentences annotated with entities and relations at least one relation. There are three types of entities: person (1685), location (1968), and organization (978); in addition there is a fourth type others (705), which indicates that the candidate entity is none of the three types. There are five types of relations: located in (406) indicates that one location is located inside another location, work for (394) indicates that a person works for an organization, OrgBased in (451) indicates that an organization is based in a location, live in (521) indicates that a person lives at a location, and kill (268) indicates that a person killed another person. There are 17007 pairs of entities that are not related by any of the five relations and hence have the NR relation between them which thus significantly outnumbers other relations.
DS7		IJCNLP 2011-New York Times (NYT)^6^	NYT contains 150 business articles from NYT. There are 536 instances (208 positive, 328 negative) with 140 distinct descriptors in NYT dataset.
DS8		IJCNLP 2011-Wikipedia^8^	Wikipedia personal/social relation dataset was previously used in Culotta et al. [6]. There are 700 instances (122 positive, 578 negative) with 70 distinct descriptors in Wikipedia dataset.

DS9	Experiment 3: task-based evaluation	Reuters 21,578^7^	Reuters-21,578 contains 21,578 documents (12,902 are used) categorized to 10 categories.
DS10		20 Newsgroups^8^	20 newsgroups dataset contains 20,000 documents (18,846 are used) categorized to 20 categories.

^1^Available at http://www.cs.cmu.edu/~mfaruqui/suite.html.

^2^Available at http://research.microsoft.com/en-us/downloads/.

^3^Available at http://corpus.byu.edu/bnc/.

^4^Available at http://cental.fltr.ucl.ac.be/team/~panchenko/sre-eval/sn.csv.

^5^Available at http://l2r.cs.uiuc.edu/~cogcomp/Data/ER/conll04.corp.

^6^Available at http://www.mysmu.edu/faculty/jingjiang/data/IJCNLP2011.zip.

^7^Available at http://mlr.cs.umass.edu/ml/datasets/Reuters-21578+Text+Categorization+Collection.

^8^Available at http://www.csmining.org/index.php/id-20-newsgroups.html.

**Table 4:** The results of the comparison of the correlation coefficient between human judgment with some relatedness measures and the proposed semantic relatedness measure.

Measure	Relevance correlation with M&C
Distance-based measures	
Rada	0.688%
Wu and Palmer	0.765%
Information-based measures	
Resnik	0.77%
Jiang and Conrath	0.848%
Lin	0.853%
Hybrid measures	
T. Hong and D. smith	0.879%
Zili Zhou	0.882%
Information/feature-base measures	
The proposed MFsSR	0.937%

**Table 5:** The results of the comparison of the accuracy and acceptance rate between the proposed semantic relatedness measure and Islam and Inkpen [7].

MRPC dataset	Relatedness threshold	Human judgment (TP + FN)	Islam and Inkpen [7]		The proposed MFsSR	
			Acc	AR	Acc	AR
Training subset (4,076)	0.1	2,753 true	0.67	1	0.68	1
	0.2		0.67	1	0.68	1
	0.3		0.67	1	0.68	1
	0.4		0.67	1	0.68	1
	0.5		0.69	0.98	0.68	1
	0.6		0.72	0.89	0.68	1
	0.7		0.68	0.78	0.70	0.98
	0.8		0.56	0.4	0.72	0.86
	0.9		0.37	0.09	0.60	0.49
	1		0.33	0	0.34	0.02

Test subset (1,725)	0.1	1,147 true	0.66	1	0.67	1
	0.2		0.66	1	0.67	1
	0.3		0.66	1	0.67	1
	0.4		0.66	1	0.67	1
	0.5		0.68	0.98	0.67	1
	0.6		0.72	0.89	0.66	1
	0.7		0.68	0.78	0.66	0.98
	0.8		0.56	0.4	0.64	0.85
	0.9		0.38	0.09	0.49	0.5
	1		0.33	0	0.34	0.03

**Table 6:** The results of the C-SynD algorithm based on the MFsSR compared to the PMI and ICA for detecting the closest synonym.

Datasets	PMI			ICA			C-SynD		
	Precision	Recall	*F*-measure	Precision	Recall	*F*-measure	Precision	Recall	*F*-measure
SN	60.6%	60.6%	0.61%	79.5%	71.6%	75.3%	85%	80%	82%
BNC	74.5%	67.9%	71%	76%	67.8%	72%	82%	71.9%	77%

**Table 7:** The results of the IOAC-QL algorithm based on AVWs compared to CRFs and TFICF approaches.

Datasets	CRFs			TFICF			IOAC-QL		
	Precision	Recall	*F*-measure	Precision	Recall	*F*-measure	Precision	Recall	*F*-measure
TREC	75.08%	60.2%	66.28%	89.3%	71.4%	78.7%	89.8%	88.1%	88.6%
BLESS	73.04%	62.66%	67.03%	73.8%	69.5%	71.6%	95.0%	83.5%	88.9%
NYT	68.46%	54.02%	60.38%	86.0%	65.0%	74.0%	90.0%	74.0%	81.2%
Wikipedia	56.0%	42.0%	48.0%	64.6%	54.88%	59.34%	70.0%	44.0%	54.0%

**Table 8:** The results of graph-based approach compared to term-based and concept-based approach using *K*-means and the HAC clustering algorithms.

Datasets	Algorithm	Term-based	Concept-based	Graph-based
		FC	FC	FC
Reuters-21578	HAC	73%	85.7%	90.2%
	*K*-means	51%	84.6%	91.8%

20 Newsgroups	HAC	53%	82%	89%
	*K*-means	48%	79.3%	87%
